# Function of the RNA-targeting class 2 type VI CRISPR Cas system of *Rhodobacter capsulatus*

**DOI:** 10.3389/fmicb.2024.1384543

**Published:** 2024-04-29

**Authors:** Jonas Kretz, Janek Börner, Tobias Friedrich, Matthew McIntosh, Tara Procida-Kowalski, Florian Gerken, Jochen Wilhelm, Gabriele Klug

**Affiliations:** ^1^Institute of Microbiology and Molecular Biology, Justus-Liebig-University, Giessen, Germany; ^2^Institute of Biochemistry, Justus-Liebig-University, Giessen, Germany; ^3^Biomedical Informatics and Systems Medicine, Justus-Liebig-University, Giessen, Germany; ^4^Institute for Lung Health, Justus-Liebig-University, Giessen, Germany

**Keywords:** CRISPR, *cas13a*, *Rhodobacter*, RNase, stress response, stationary phase, transcriptome

## Abstract

Bacteria use CRISPR Cas systems to defend against invading foreign nucleic acids, e.g., phage genomes, plasmids or mobile genetic elements. Some CRISPR Cas systems were reported to have physiological importance under a variety of abiotic stress conditions. We used physiological tests under different stress conditions and RNA-seq analyses to address the possible function of the RNA-targeting class 2 type VI CRISPR Cas system of the facultative phototrophic α-proteobacterium *Rhodobacter capsulatus*. Expression of the system was low under exponential non-stress conditions and high during oxidative stress, membrane stress and in stationary phase. Induction of the CRISPR Cas system in presence of a target protospacer RNA resulted in a growth arrest of *R. capsulatus*. RNA-seq revealed a strong alteration of the *R. capsulatus* transcriptome when *cas13a* was induced in presence of a target protospacer. RNA 5′ end mapping indicated that the CRISPR Cas-dependent transcriptome remodeling is accompanied by fragmentation of cellular RNAs, e.g., for mRNAs originating from a genomic locus which encodes multiple ribosomal proteins and the RNA polymerase subunits RpoA, RpoB and RpoC. The data suggest a function of this CRISPR Cas system in regulated growth arrest, which may prevent the spread of phages within the population.

## 1 Introduction

**Figure 1:**
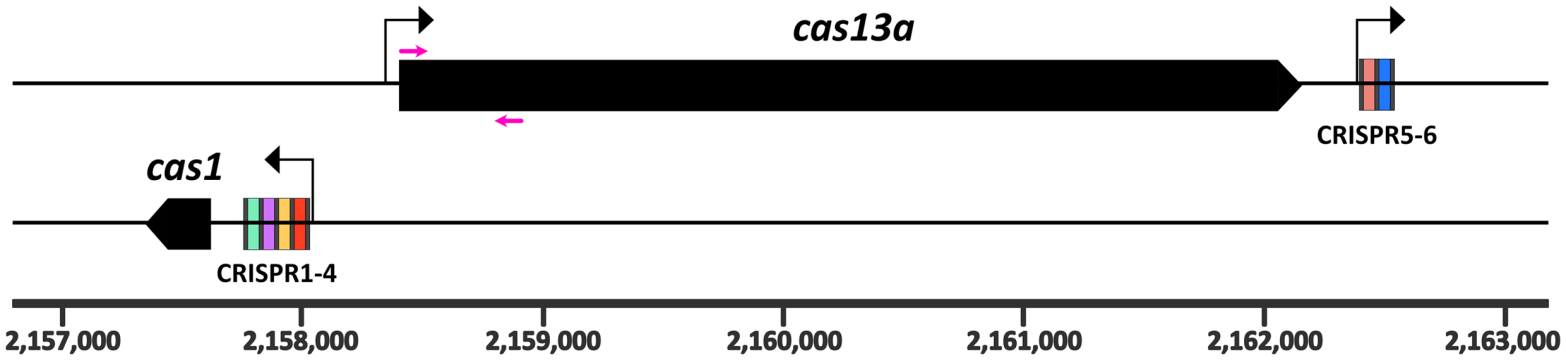
Genomic arrangement of the class 2 type VI CRISPR Cas locus on the *R. capsulatus* SB1003 chromosome. The *cas13a* (plus strand) and *cas1* (negative strand) genes are shown as black boxes. The *cas1* gene is preceded by the larger CRISPR array, containing four spacer regions. A second CRISPR array is located directly downstream of the *cas13a* gene and contains only two spacers (5–6). Repeats are indicated as gray bars. The six spacer regions are highlighted in different colors. The chromosomal coordinates are displayed at the bottom. Three promoters of the system are indicated as black arrows. Primers used for the homologous recombination constructs are marked as magenta arrows and their sequences are listed in Supplementary Table S2. Methodological details are provided as Supplementary material.

In their natural environment bacteria are exposed to numerous different stress conditions. They have to cope with, e.g., temperature changes, UV radiation, limitation of nutrients, drought, or oxidative stress. Additionally, phototrophic and facultative phototrophic bacteria like *Rhodobacter capsulatus* face the problem of photooxidative stress mainly caused by singlet oxygen, which is generated when bacteriochlorophyll, light and oxygen are present simultaneously. In the past we have elucidated the molecular mechanisms that allow *R. capsulatus* or *Rhodobacter sphaeroides*, now renamed to *Cereibacter sphaeroides* (Hördt et al., 2020), to cope with oxidative and photooxidative stress, with iron limitation, and with nutrient starvation (Li et al., 2003, 2004; Glaeser and Klug, 2005; Berghoff et al., 2009; Nuss et al., 2009; Remes et al., 2014; Müller et al., 2016; Bathke et al., 2019; McIntosh et al., 2019; Licht et al., 2020; Eisenhardt et al., 2021). A global transcriptome analysis of *R. capsulatus* revealed increased expression of a class 2 type VI CRISPR Cas system (Figure 1) in response to singlet oxygen (Licht et al., 2020).

CRISPR Cas systems are a main protective mechanism of bacteria against phages that are prevalent in nature (Barrangou et al., 2007). Although the numbers vary across ecosystems, it is estimated that the ratio of bacterial cells to phage particles is typically around 1:10 in many natural environments (Mushegian, 2020; Chevallereau et al., 2022). Therefore, an effective protection against phage infection is crucial for bacterial survival. CRISPR Cas systems are considered as the “adaptive immune system” of bacteria, since they prevent recurring phage infection by specific degradation of the respective phage genome (Hille and Charpentier, 2016). Upon first infection with a virus, its genome can be degraded and pieces of the genome can then be integrated into the bacterial chromosome in the form of spacers between palindromic repeats forming the CRISPR arrays (Brouns et al., 2008). The mature CRISPR RNAs (crRNAs; processing product consisting of one spacer and one repeat sequence) are mandatory to bind to a specific recognition sequence, the protospacer in the phage genome, leading to an activation of nucleolytic activity of the Cas-crRNA complex (Jackson et al., 2017).

Class 2 type VI CRISPR Cas systems target RNA, and the single effector nuclease belongs to the Cas13 protein family (divided into the four major subgroups Cas13a-d). Compared to other CRISPR Cas systems, e.g., the dsDNA-targeting Cas9 systems, class 2 type VI systems are relatively rare in bacteria (Makarova et al., 2020; Adler et al., 2022). In our study, we address the role of the class 2 type VI CRISPR Cas system in the stress response of the facultative phototrophic α-proteobacterium *R. capsulatus*. The single effector protein Cas13a has two higher eukaryotic and prokaryotic nucleotide binding (HEPN) domains, which are mandatory for RNA binding and ribonucleolytic activity. Interestingly, bacterial HEPN domains, except the ones found in CRISPR Cas systems, are related to toxin-antitoxin (TA) systems or abortive infection (Abi) systems. Therefore, it seems likely that HEPN domain-containing Cas effectors, like Cas13a of *R. capsulatus*, may have evolutionarily developed from TA or Abi systems (Koonin and Makarova, 2017). Abi systems usually sense RNA or proteins from phages, which signal an advanced or almost finished reproductive cycle of the phage (Lopatina et al., 2020). After activation, Abi systems can either lead to a strong reversible growth arrest of the bacteria or can go as far as cell death and lysis. Both ways ultimately stall or stop phage propagation in the bacterial community and therefore, Abi is also referred to as “altruistic suicide” (Koonin et al., 2017). CRISPR-associated Abi has already been shown for some type III systems (Mayo-Muñoz et al., 2022; Steens et al., 2022). In these systems the target RNA is recognized by the crRNA-Cas complex and gets degraded together with the origin DNA (Foster et al., 2020). Besides this specific degradation, the type III CRISPR-associated HEPN domain-containing ribonuclease Csm6 gets activated and consequently cleaves both host and phage RNAs leading to growth arrest (Makarova et al., 2014; Rostøl and Marraffini, 2019; Garcia-Doval et al., 2020).

Recently the crystal structure of *Rc*Cas13a has been fully solved. The authors further showed that *Rc*Cas13a is mandatory for processing of pre-crRNA into mature crRNAs, and exhibits off-target trans RNA cleavage activity in *Escherichia coli* (Kick et al., 2022). No studies on this system have been performed in *R. capsulatus* to date, thus the physiological role of the class 2 type VI CRISPR Cas system in *R. capsulatus* has not been elucidated yet. Previous studies on Cas13a from *Listeria seelegeri* revealed that the class 2 type VI system, once it is activated by target RNA binding, degrades various RNAs in *cis* and *trans* manner (Meeske et al., 2020) as it was also shown for the recombinantly expressed *Rc*Cas13a-crRNA complex in *E. coli* (Kick et al., 2022). In *L. seelegeri*, this activation leads to a dormant cell state diminishing phage propagation in the bacterial population as well as decreasing sensitivity against certain stress conditions (Meeske et al., 2020).

In this study, we show that expression of the Cas13a and CRISPR regions of the class 2 type VI system from *R. capsulatus* is induced by various stress conditions. RNA-seq revealed that simultaneous expression of the CRISPR Cas system and a target protospacer RNA led to a drastically altered RNA abundance profile, and is accompanied by a severe growth arrest. RNA-seq based RNA 5′ end mapping revealed that under these conditions indeed a high amount of novel RNA 5′ ends are generated in the *R. capsulatus* transcriptome, especially in a genomic locus consisting of many genes for ribosomal proteins.

## 2 Materials and methods

### 2.1 Bacterial growth conditions

*Rhodobacter capsulatus* SB1003 (Yen and Marrs, 1976) was used as wild type strain in this study. All *Rhodobacter* strains used and created in this study are listed in Supplementary Table S1. The *E. coli* strain S17-1 (Simon et al., 1986) was used for biparental conjugation on peptone yeast (PY) agar plates (10% tryptone/peptone from casein, 0.5% yeast extract, 2 mM CaCl_2_, 2 mM MgCl_2_, 1.5% agar) (Klug and Drews, 1984). Further selection of conjugants was performed on malate minimal (Remes et al., 2014) agar plates containing either 1 μg/mL tetracycline (pPHU plasmids), 25 μg/mL kanamycin (pK plasmids) or 10 μg/mL gentamycin (pCV2 plasmids). For liquid cultures *R. capsulatus* was cultivated in malate minimal medium under microaerobic conditions (25–30 μM dissolved oxygen) in the dark at 32°C. For growth at high oxygen tension, *R. capsulatus* was cultured in presence of 160–180 μM dissolved oxygen. The genetically engineered strains for altered *cas13a* expression were grown with either 0.1 μM crystal violet (repression of *cas13a*) or with addition of 0.5 mM IPTG (induction of *cas13a*). Overnight cultures of these strains used for inoculation were always cultivated with 0.1 μM crystal violet.

### 2.2 Primers, plasmids, and strain constructions

All primers used in this study are listed in Supplementary Table S2 as well as all plasmids in Supplementary Table S3. Cloning procedures of genetical constructs and generation of mutant strains are explained in detail in the Supplementary material.

### 2.3 Fluorescence measurements

Fluorescence measurements were performed using 100 μL of the pPHU carrying cultures in transparent 96-well plates (Greiner bio one) in an Infinity plate reader (Tecan). Optical density was measured at λ = 660 nm and fluorescence intensity of mVenus was measured using excitation at 515 nm and emission of 548 nm.

### 2.4 Western blot analysis

For Western Blot analysis, cells were harvested and normalized to the optical density. After centrifugation the pellet was resuspended in water and SDS sample buffer (500 mM Tris pH 6.8, 10% SDS w/v, 20% Glycerol v/v, 0.05% bromophenol blue w/v). The samples were heated to 95°C for 10 min and vigorously mixed on the vortex for at least 1 min. To remove debris, the samples were again centrifuged (10 min, 13,000 rpm) and the supernatant was loaded into wells of the SDS-PAGE gel. After semidry blotting to a PVDF membrane (Roth; 0.45 μm), protein detection was performed using a monoclonal mouse ANTI-FLAG M2-Peroxidase (HRP) antibody from Sigma-Aldrich. The SuperSignalTM West Pico PLUS Chemiluminescent Substrate Kit (Thermo Fisher) was used to develop the western blot signals.

### 2.5 Survival assay

*R. capsulatus* cultures were diluted at indicated time points up to 10^−6^. 100 μL of this dilution were plated on malate minimal agar plates containing 0.1 μM crystal violet. The plates were incubated in the dark at 32°C for 48 h and colony forming units were counted afterwards. These experiments were performed with biological triplicates as well as technical duplicates and two different dilutions were plated per strain and condition to maximize reliability of the data.

### 2.6 Zone of inhibition assay

Exponentially growing *R. capsulatus* cultures were normalized per cell density, mixed with malate minimal soft agar (0.5% agar w/v) and subsequently poured on 15 mL of malate minimal agar plates. After 10 min, filter papers were added at the center of the plate and coated with 5 μL water as negative control, or 5 μL stress inducing agents as indicated (1 M H_2_O_2_, 5% SDS w/v + 1 mM EDTA, or 10 mM methylene blue). Afterwards, the plates were incubated in the dark at 32°C for 48 h. Only plates for testing singlet oxygen sensitivity were incubated under 800 W/m^2^ white light. After incubation, the diameter of the growth inhibited zones were measured.

### 2.7 RNA isolation

For RNA sequencing, *R. capsulatus* cultures were grown for 6 h under microaerobic conditions, starting at OD_660_ ≈ 0.1. For northern blot analysis, cell cultures were grown under microaerobic conditions to exponential phase (6 h) or indicated time points. Outgrowth was performed by reinoculation (OD_660_ ≈ 0.1) of fresh medium 20 or 90 min prior to collecting the samples. Cells were sedimented via centrifugation at 10,000 rpm at 4°C for 10 min. Afterwards, RNA was extracted using the hot phenol method (Janzon et al., 1986; Damm et al., 2015) as described previously (Börner et al., 2023a). For RNA-seq, residual DNA contaminations were degraded using the TURBO DNA-*free* Kit (Invitrogen) as described in the manufacturer’s manual. The sequencing libraries were constructed as described previously (Grützner et al., 2021a), using the NEBNext Multiplex Small RNA Library Prep Set for Illumina (NEB).

### 2.8 Northern blot analysis

For northern blot analysis, 5 μg of total RNA were electrophoretically separated on denaturing 10% polyacrylamide gels. Following electrophoresis, RNAs were transferred to a nylon membrane (pore size 0.45 μm) via semidry electroblotting and subsequently immobilized by UV crosslinking. Radiolabeled deoxyoligonucleotides complementary to the CRISPR1-4 RNA or 5S rRNA were used as probes for signal generation via phosphor imaging. [γ-^32^P]-ATP (Hartmann Analytic) was used for 3′ end radiolabeling reactions with T4 polynucleotide kinase (NEB) following the manufacturer’s protocol. 5S rRNA was used for quantification. Radiolabeled probes were hybridized with membrane immobilized RNAs under low stringency conditions using the Church buffer system (Church and Gilbert, 1984). For removal of background signals, membranes were washed twice with 5x SSC buffer containing 0.01% SDS (w/v) for 5 min at 42°C. Subsequently, membranes were air-dried and exposed to phosphor imaging screens (Bio-Rad) for signal generation. For removal of hybridized probes, the membranes were incubated in 5x SSC buffer containing 0.1% SDS (w/v) for 20 min at 95°C and 80 rpm.

### 2.9 Quantitative reverse transcriptase PCR

Quantitative reverse transcriptase PCR (qRT-PCR) of DNA-free total RNA was performed as described recently (Börner et al., 2023a) using the Brilliant III Ultra-Fast SYBR Green QRT-PCR Master Mix kit (Agilent Technologies) according to the manufacturer’s manual. As reference for normalization, 1 ng of a previously generated *in vitro* spike-in transcript (*sinI* from *Sinorhizobium meliloti*) (Grützner et al., 2021b) was added per 10 μg of DNA-free total RNA sample. The used primers specific for a ~ 200 bp fragment of *rplJ*, *rplP*, *rplF* and the spike-in transcript (*sinI*) are listed in Supplementary Table S2. Relative RNA abundances were calculated from independent biological triplicates, each in technical replicates, using the Pfaffl quantification model (with primer efficiency correction) (Pfaffl, 2001).

### 2.10 RNA-seq data processing

Bioinformatics analysis was described in detail recently (Börner et al., 2023b). In summary, raw sequencing reads were adapter and quality trimmed using TrimGalore v.0.6.10^1^ and subsequently aligned against the *Rhodobacter capsulatus* SB1003 reference genome (version ASM2186v1) using the READemption pipeline v.1.0.5 (Förstner et al., 2014) and stored as binary alignment maps (BAM). Coverage tracks for each file were generated using the *coverage* function of the READemption pipeline and stored as .wig files. Merged .wig files were generated within R v.4.1.2. 5′ end mapping was performed by identification of the first base of each BAM file using bedtools’ *genomecov* function (Quinlan and Hall, 2010). Read counts per genes were calculated using the *summarizeOverlaps* function of the GenomicAlignments package (Lawrence et al., 2013) with the corresponding gene transfer file. Resulting read counts tables were normalized and differentially expressed genes were identified with DESeq2 v.1.32 (Love et al., 2014). PCA plots were generated based on DESeq2 results.

## 3 Results and discussion

### 3.1 Stress conditions and stationary phase activate the promoters of the class 2 type VI CRISPR Cas system

**Figure 2:**
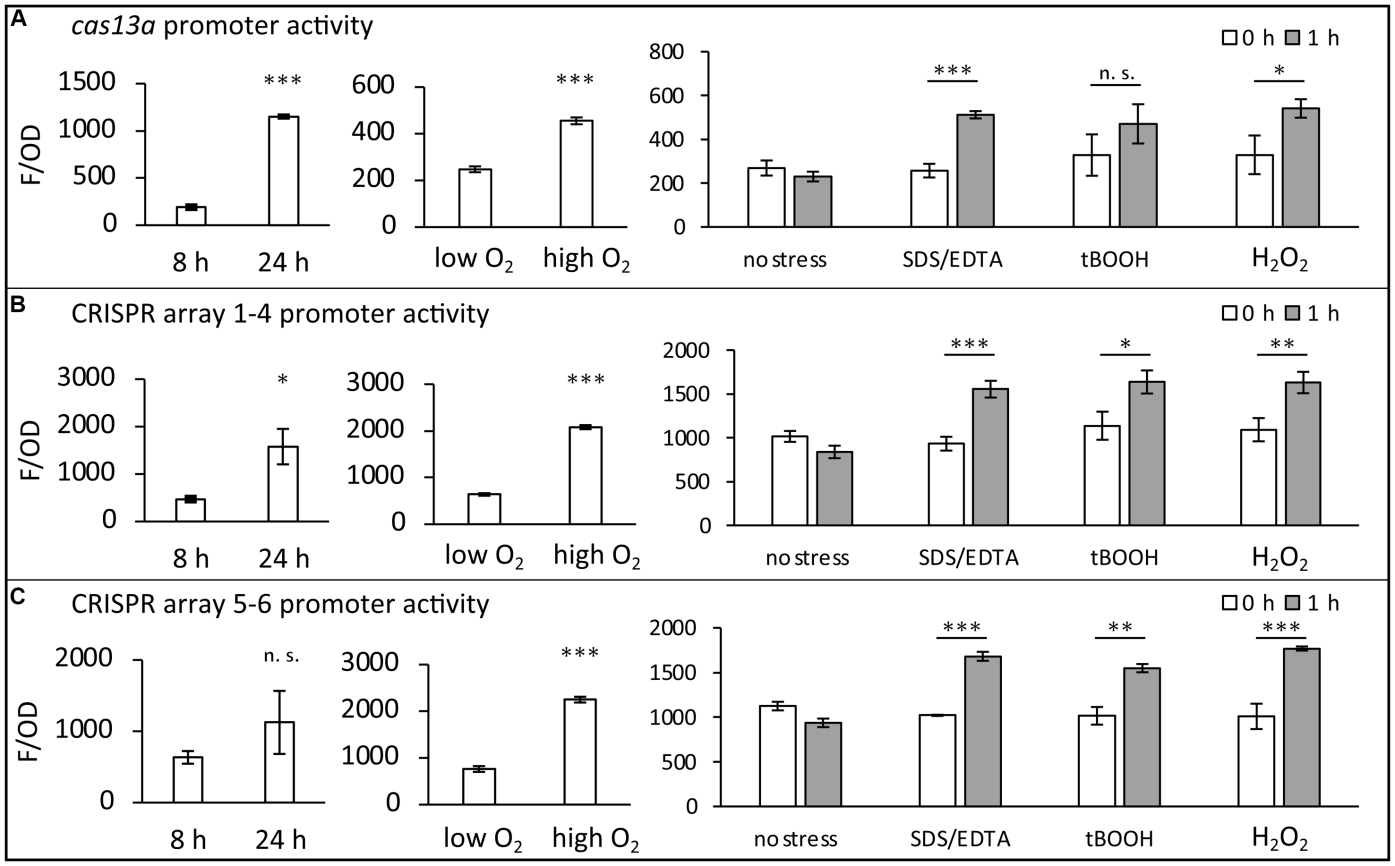
Promoter activities within the class 2 type VI CRISPR Cas system of *R. capsulatus*. Transcriptional mVenus reporter fusions were used to investigate the activity of three promoters under the indicated conditions *in vivo.* **(A)** Fusion of the *cas13a* preceding promoter region to the mVenus ORF. **(B)** Fusion of the larger CRISPR array preceding promoter region to the mVenus ORF. **(C)** Fusion of the smaller CRISPR array preceding promoter region to the mVenus ORF. Fluorescence intensities of wild type cultures carrying a plasmid with one of the transcriptional fusion constructs were measured after 8 h (exponential phase) or 24 h (stationary phase) of cultivation. Additionally, promoter activities of exponentially growing cultures in the presence of either 0.3 mg/L dissolved oxygen (low oxygen) or 7.8 mg/L dissolved oxygen (high oxygen) were analyzed. To investigate the stress response, 0.005% (w/v) SDS + 1 mM EDTA, 1 mM tBOOH or 10 mM H_2_O_2_ was added to exponentially growing cultures (0 h) for 1 h. The fluorescence intensities (F) were normalized to the optical density at 660 nm (OD). The mean value and standard deviation of independent biological triplicates is plotted. Student’s two-sided t-test was used to assess the statistical significance of the difference in mean values (n. s., not significant; * *p*-value <0.05; ** *p*-value <0.01; *** *p*-value <0.001).

In a previous RNA-seq analysis we observed increased transcript levels of *cas13a* and the CRISPR RNAs in presence of methylene blue, a singlet oxygen producing photosensitizer (Licht et al., 2020) (NCBI GEO accession number GSE134200). To investigate under which conditions the class 2 type VI system of *R. capsulatus* is transcriptionally active, we used transcriptional fusions of its promoters with the gene for the fluorescent protein mVenus. To identify these promoters, we used an approach similarly to that previously used to identify promoters in *R. sphaeroides* (Remes et al., 2017). Firstly, inspection of the RNA sequencing profiles (e.g., read coverage visualized with the Integrated Genome Browser) indicated possible transcription starts where the number of RNA reads dramatically increased at a mapped location of the genome. Secondly, the presence of conserved promoter elements, such as TTG at position-35 of the transcription start, was also taken as a hint. On this basis, the approximately 300 bp region was selected for cloning into the transcriptional fusions with the mVenus reporter gene. As a routine procedure for a negative control, a construct carrying a promoterless mVenus gene was included. A detailed description of the transcriptional fusion constructs is provided in the Supplementary material. The results indicate that the promoters of the two CRISPR arrays and the main ORF, encoding the protein Cas13a (schematically shown in Figure 1), are closely co-regulated and activated under several stress conditions. The highest increase of promoter activities was observed during stationary growth phase, 24 h after inoculation of fresh medium with an over-night culture (24 h) compared to exponential phase (8 h) (Figures 2A–C). Yet, certain stress conditions also affect promoter activities in the exponential phase. While addition of SDS/EDTA, or peroxides in form of tBOOH (tertiary butyl alcohol, organic peroxide) or H_2_O_2_ led to a 1.5–2-fold increase in promoter activities, growth at high concentrations of dissolved oxygen led to the strongest increase in promoter activities (up to ~6 fold compared to growth at low oxygen conditions). In nature, organic hydroperoxides stem from interactions of cellular macromolecules with singlet oxygen, which is generated during the simultaneous presence of chlorophyll, light and oxygen (Glaeser et al., 2011). We assume that the close co-regulation of *cas13a* and the CRISPR arrays, as seen in our transcriptional reporter data, is beneficial for *R. capsulatus*, as processing by Cas13a is a crucial step during the maturation of crRNAs. Stress conditions may facilitate phage attack. Especially membrane damage is linked to phage adhesion (Ratner et al., 2015). The increased CRISPR/Cas levels during stress conditions may prepare the bacteria against potential phage attacks.

**Figure 3:**
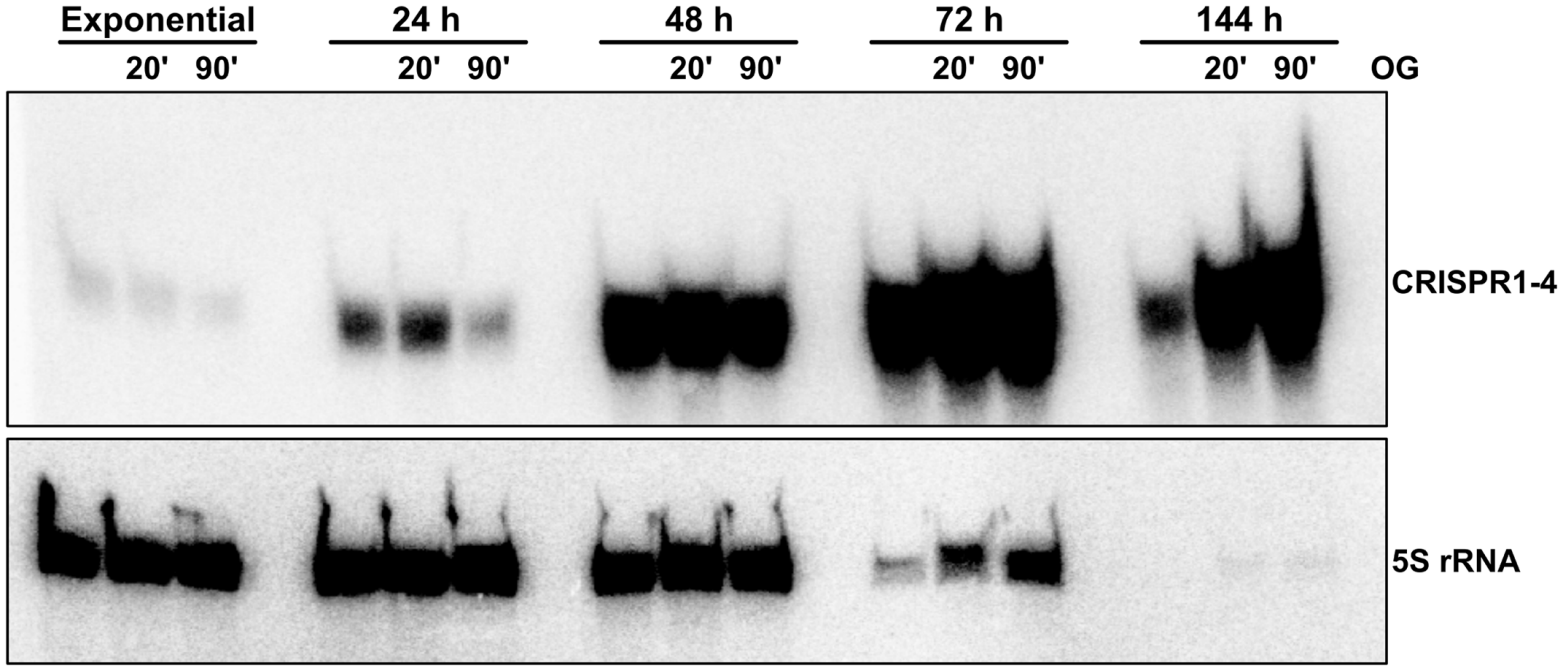
CRISPR 1–4 RNA accumulates in late growth phases. Total RNA isolated from *R. capsulatus* wild type cultures at different stages of growth (hours after inoculation from overnight cultures) was analyzed by northern blot. 5 μg of total RNA per lane were electrophoretically separated on denaturing 10% (w/v) polyacrylamide gels. Radiolabeled deoxyoligonucleotides with complementary sequence to 5S rRNA or CRISPR1-4 RNA were used as probes for signal generation by phosphor imaging. OG (outgrowth): freshly inoculated cultures grown for 20 min or 90 min after 5 h (exponential), 24 h, 72 h, or 144 h of growth.

Furthermore, northern blot analysis revealed even higher signals for the CRISPR1-4 RNA in late stationary phase (Figure 3), as they reached a maximum after 72 h of growth. In contrast, at these late time points (up to 144 h after inoculation) the 5S rRNA, which is frequently used as a loading control for RNA from exponential phase cells, is already heavily degraded. While a steady increase in the signal of CRISPR1-4 RNA has been observed in samples taken from 24 h to 72 h, a decreased northern blot signal was obtained from total RNA samples collected 144 h after inoculation, compared to the strong signals at 48 h or 72 h. To analyze the CRISPR1-4 levels of cells resuming growth after stationary phase, 24 h, 48 h, 72 h, or 144 h after inoculation aliquots of the cultures were reinoculated into fresh medium and samples for northern blot analysis were taken at 20 min and 90 min of this outgrowth. An increase of 5S rRNA signal was visible for outgrowth after 72 h and 144 h, and also the signals for CRISPR1-4 RNA increased continuously over time. Similar results were obtained when CRISPR5-6 RNA was probed (not shown). These results indicate that CRISPR RNA levels may increase for many hours, even when cultures have stopped growing. At very late time points, CRISPR levels seem to decrease, and show a very strong increase as soon as the cells resume growth. However, definitive conclusions about increasing or decreasing RNA levels are difficult, since normalization is based on loading equal amounts of total RNA. Consequently, RNAs with unchanged levels would be enriched in samples with decreased rRNA levels. Nevertheless, the different changes in band intensities for CRISPR RNAs compared to 5S rRNA are remarkable.

As also our data of the transcriptional fusion reporters (Figure 2) pointed towards lowest transcriptional activity during exponential phase, we conclude that the enrichment of CRISPR1-4 (Figure 3) may be caused by induced transcription rates of the *R. capsulatus* class 2 type VI system during stationary growth phase. Induced expression during stationary phase was also observed previously for *Salmonella enterica*, *E. coli*, *Legionella pneumophila* and the haloarchaeon *Haloferax mediterranei* CRISPR Cas systems (Gunderson and Cianciotto, 2013; Li et al., 2013; Majsec et al., 2016). However, the benefit of induction of CRISPR Cas systems during stationary phase remains elusive. It has been speculated that this may help to utilize uptaken foreign nucleic acids as a nutrient source (Finkel and Kolter, 2001; Ratner et al., 2015). Starvation can also serve as a signal for prophages to become lytic (Hansen et al., 2005), and therefore activation of CRISPR Cas systems could protect the bacterial population. The strongly increased expression of the CRISPR Cas system during outgrowth could well protect the cells when they leave a state that does not favor phage propagation.

In *Rhodobacter*, the alternative sigma factors RpoE, RpoHI, and RpoHII have important roles in the response to oxidative and photooxidative stress (Anthony et al., 2005; Green and Donohue, 2006; Berghoff et al., 2009; Nuss et al., 2010; Licht et al., 2020; Eisenhardt et al., 2021). RpoHI and RpoHII are also important for the adaptation to stationary phase (Remes et al., 2017; McIntosh et al., 2019). *Rhodobacter* species lack an RpoS-like master regulator (McIntosh et al., 2019). Since *cas13a* is induced by oxidative stress and stationary phase, we tested the role of these alternative sigma factors in this induction. Mutants of *rpoE*, *rpoHI*, or *rpoHII* showed *cas13a* expression in response to growth phase and stress conditions in the same range as observed for the wild type (Supplementary Figure S1). This excludes a major role of these sigma factors in regulation of the CRISPR array and *cas13a* promoters. In *E. coli* and *S. enterica* expression of CRISPR Cas is activated by the transcriptional regulator LeuO (Hernández-Lucas et al., 2008; Westra et al., 2010; Medina-Aparicio et al., 2011; Shimada et al., 2011; Mitić et al., 2020), which is active during phosphate starvation and stationary phase (VanBogelen et al., 1996; Fang et al., 2000; Stratmann et al., 2012). A BLAST search against the *R. capsulatus* genome with LeuO from *E. coli* as query revealed three protein candidates with homology to LeuO: RCAP_RS16295, RCAP_RS11235 and RCAP_RS02120. When we compared RNA-seq data of *R. capsulatus* samples from exponential and stationary phase, no change in expression was visible for either of the three candidates (not shown). Therefore, the mechanisms of activation of the CRISPR and *cas* promoters require further investigations. E.g. sRNAs were shown to make important contributions to the regulated formation of the photosynthetic apparatus and to regulation of stress responses in *R. sphaeroides* (Mank et al., 2012; Adnan et al., 2015; Billenkamp et al., 2015; Müller et al., 2016; Peng et al., 2016; Eisenhardt et al., 2018, 2021; Reuscher and Klug, 2021; Spanka et al., 2022). Many sRNAs were detected in the *R. capsulatus* transcriptome (Licht et al., 2020), which are still awaiting functional characterization.

### 3.2 Effect of Cas13a expression on stress resistance and survival

To investigate effects of an altered Cas13a level *in vivo*, we chromosomally modified the *cas13a* locus in *R. capsulatus* by inserting an inducible promoter directly upstream of the *cas13a* gene, enabling tight transcriptional control of *cas13a* expression by addition of subinhibitory crystal violet concentrations (strong repression of *cas13a*) or IPTG (strong induction of *cas13a*). The inducible promoter is a synthetic construct consisting of the 16S gene promoter from *R. sphaeroides* flanked by Lac operators. This promoter, together with the *lacI* gene from *E. coli*, was previously tested in *R. capsulatus* (Kretz et al., 2023) and found to have at least a 100-fold induction capacity in gene expression. Furthermore, to observe effects of the absence of Cas13a activity *in vivo*, we generated an additional *R. capsulatus* strain by inserting a transcription terminator between the *cas13a* gene and its promoter on the chromosome. The genomic background of both modified strains is schematically shown in Supplementary Figures S2A,B. In both cases single homologous recombination of an integration cassette modifies the promoter region of the *cas13a* gene. While the *cas13a^–^ strain* is lacking a promoter directly upstream of *cas13a*, the inducible *cas13a* strain is characterized by substitution of the native promoter with an IPTG inducible promoter. Western blot analysis verified that in this strain, Cas13a is only produced after addition of IPTG (Supplementary Figure S2). To exclude growth inhibiting effects of the genetic modifications, we monitored the growth behavior of the wild type and the engineered strains with altered amounts of Cas13a over a time period of 56 h by photometric measurement of the optical density (λ = 660 nm). Notably, no pronounced effects of the modification on growth were visible (Supplementary Figure S3), indicating that Cas13a is dispensable for growth of *R. capsulatus* under non-stress conditions.

To further investigate whether the alteration of Cas13a levels affects growth under stress conditions, we performed zone of inhibition assays with the modified strains and the wild type as control. Therefore, we spotted droplets of H_2_O_2_, methylene blue (a singlet oxygen producing photosensitizer) (DeRosa and Crutchley, 2002) or SDS/EDTA on *R. capsulatus* containing soft agar plates. Following an incubation of the plates at 32°C, the diameter of the resulting zones of growth inhibition were measured. As observed for growth under non-stress conditions (Supplementary Figure S3), no severe effects of the modified Cas13a levels on the stress resistance/sensitivity were visible, as seen in similarly sized zone of inhibitions between the modified strains and the wild type control. These data suggest that Cas13a itself has no major function in stress resistance of *R. capsulatus* against H_2_O_2_ or singlet oxygen-induced oxidative stress and SDS/EDTA-induced membrane stress. While elevated expression of *cas2* led to decreased survival rates of *Mycobacterium smegmatis* under oxidative stress (Huang et al., 2016), deletion of *cas6* resulted in reduced oxidative stress resistance in *Mycobacterium bovis* (Yang et al., 2021). In the intracellular pathogen *Francisella novicida*, a *cas9*-dependent CRISPR Cas system enhances membrane integrity by conferring resistance against membrane damage-inducing compounds (Sampson et al., 2014). Furthermore, in *Streptococcus mutans* an increased sensitivity against detergent-induced membrane stress as well as superoxide- and H_2_O_2_-induced oxidative stress was observed for mutants of a class 2 type II CRISPR Cas system, but not for mutants of a class 1 type I system (Serbanescu et al., 2015).

### 3.3 Presence of a protospacer sequence activates Cas13a and leads to growth arrest

**Figure 4:**
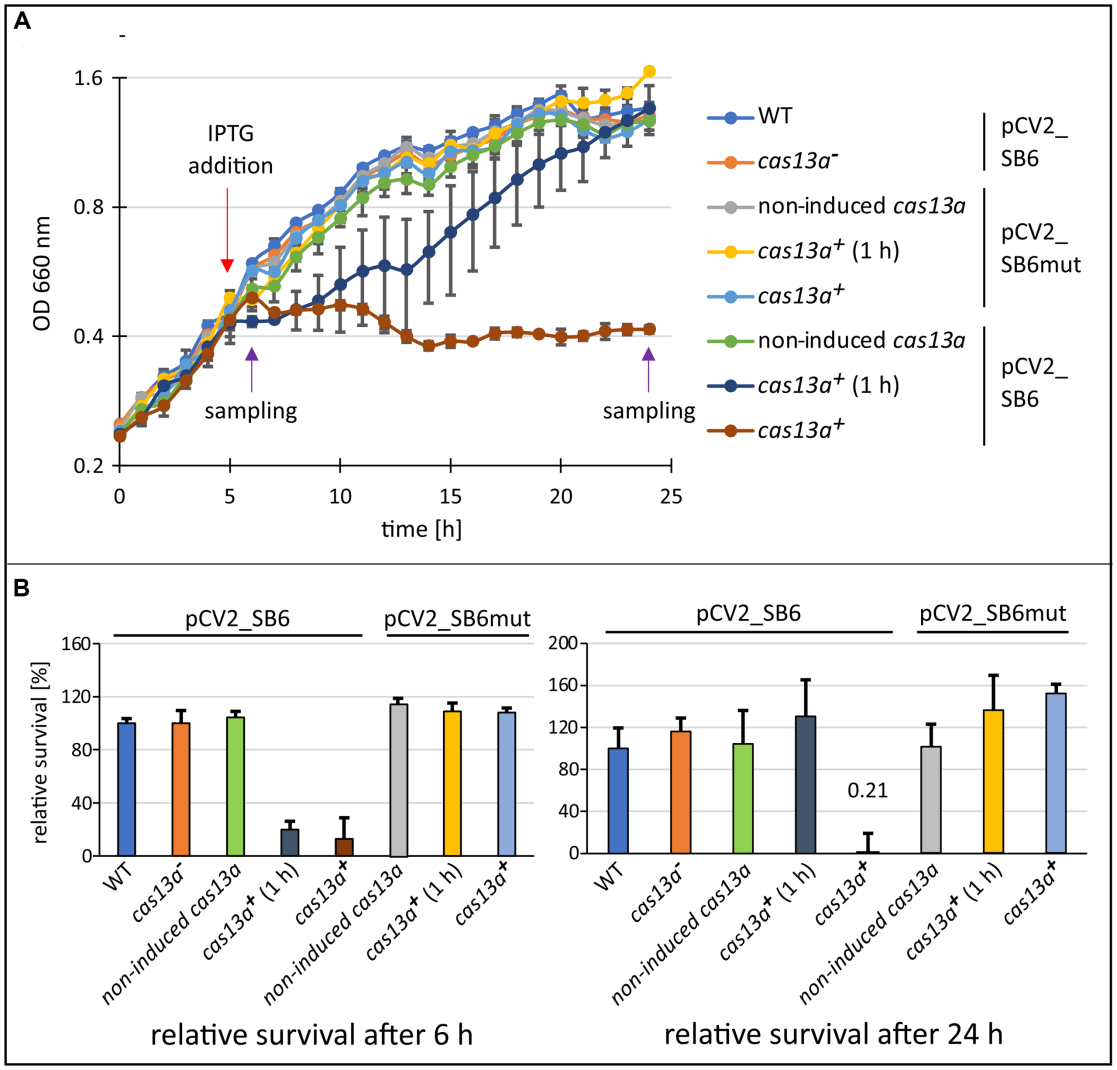
The induction of *cas13a* in presence of protospacer RNA leads to severe inhibition of growth. **(A)** Growth effect of inducing *cas13a* expression and simultaneous protospacer expression. The growth behavior of the *cas13a*^***−***^ strain, the inducible strain without IPTG-inductor (non-induced *cas13a*), after short induction with IPTG for 1 h and subsequent removal of IPTG (*cas13a*^***+***^ 1 h), or continuous induction with IPTG (*cas13a*^***+***^) was monitored over 24 h. The strains were carrying either plasmid pCV2_SB6mut (protospacer 6 sequence harboring a point mutation) or pCV2_SB6 (fully intact protospacer 6 sequence). Purple arrows indicate time points of sampling for the subsequent survival assay shown in panel (B). **(B)** Survival assay for determination of colony forming units after 6 h and 24 h of growth. Cell culture samples collected at indicated time points (left: 6 h samples; right: 24 h samples) were diluted and plated on malate minimal medium plates. Data is visualized as survival rate relative to the native *R. capsulatus* SB1003 strain. Mean value and standard deviation of biological triplicates are shown.

Since activation of the *R. capsulatus* Cas13a effector was previously shown to be dependent on the binding of the protospacer to the Cas13a-crRNA complex (Kick et al., 2022), we decided to construct an extrachromosomal plasmid (pCV2_SB6), which allows inducible expression of a protospacer RNA, to further investigate the physiological consequences of altered Cas13a expression in presence of a protospacer RNA *in vivo*. As a control construct, also an additional plasmid carrying a protospacer sequence with a single nucleotide exchange was cloned (pCV2_SB6mut). Following cloning procedures in *E. coli*, we used diparental conjugation to transfer the plasmids into the wild type or strains with altered *cas13a* expression. For phenotypic characterization, we monitored the growth and quantified colony forming units of the resulting conjugants over a time span of 24 h (Figure 4).

Once transcription of *cas13a* and of the extrachromosomally expressed target RNA are induced by the addition of IPTG 5 h after inoculation, *R. capsulatus* halted its growth (Figure 4A, brown data points). A similar effect was also reported recently for *E. coli* recombinantly expressing the *R. capsulatus* class 2 type VI system (Kick et al., 2022). Our data show that this growth arrest is reversible, since *R. capsulatus* cultures resumed growth after removal of the IPTG-inductor (Figure 4A, dark blue data points). Moreover, we observed that this mechanism appears to be highly specific, as a single nucleotide exchange in the protospacer sequence caused abolishment of the growth inhibiting effect (Figure 4A, yellow and light blue dots). Remarkably, after removal of IPTG the variance of the data points from individual cultures is much higher than for the growth curves of the other conditions. It is conceivable that this variance is caused by a higher heterogeneity of the cells resuming growth.

**Figure 5:**
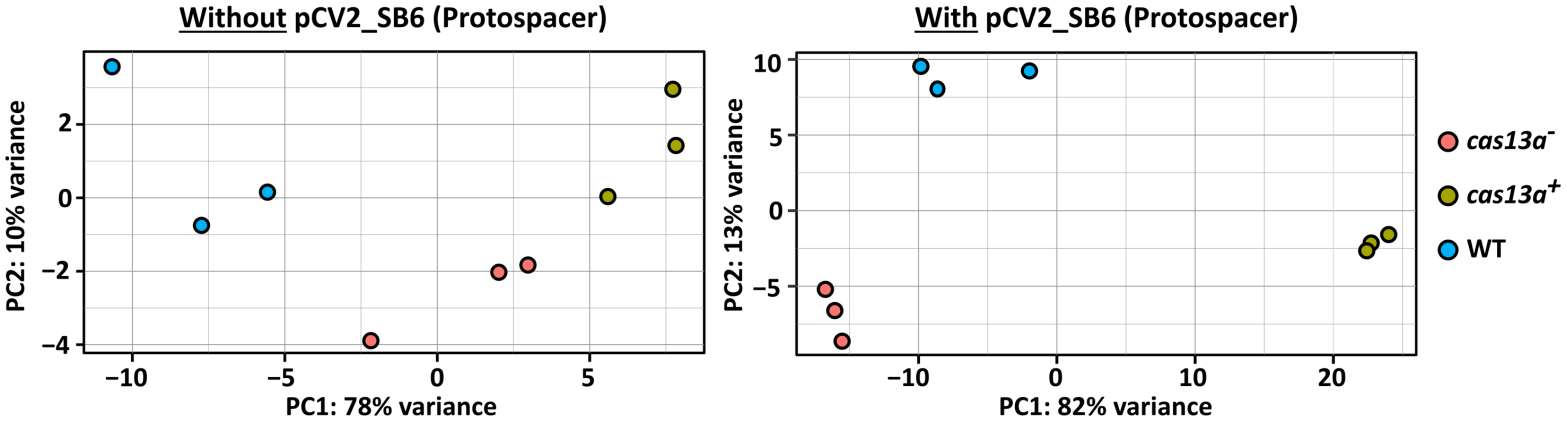
RNA-seq based transcriptome profiling reveals profound variation of transcriptomes upon simultaneous expression of protospacer RNA and *cas13a*. Principal component analysis (PCA) visualizing the variation of the transcriptomes between *R. capsulatus* wild type (blue), the mutant with induced Cas13a expression (green) and without induced Cas13a expression (red). RNA-seq data of total RNA from biological triplicates was analyzed. Strains were grown either in absence of protospacer RNA (left panel) or carrying pCV2_SB6 for ectopic expression of SB6 protospacer RNA (right panel).

Assessing the cell survival rates of culture samples taken at 6 h or 24 h (Figure 5B) indicated that the growth arrest is not accompanied by rapid cell death, as cultures which were only induced for 1 h before removing the inductor (dark blue data points), reached comparable colony forming units as the negative controls after 24 h. Similar results were also reported for *L. seeligeri*, where bacteria with an active Cas13a complex were still respiratory active during growth arrest (Meeske et al., 2020). Likely, the reason for this growth arrest are rapid changes in the transcriptome caused through the off-target ssRNA cleavage activity of Cas13a after the target RNA is bound to the Cas13a-crRNA complex (Abudayyeh et al., 2016; East-Seletsky et al., 2016; Gootenberg et al., 2017; Liu et al., 2017; Meeske et al., 2020). Such Abi systems prevent the spread of the phages to the surrounding population without killing the cells. Based on sequence analysis, it is estimated that over 70% of prokaryotes encode abortive infection systems (reviewed in Rousset and Sorek, 2023).

### 3.4 Changes of the transcriptome in presence of Cas13a and protospacer RNA

To further characterize the consequences of a simultaneous presence of the class 2 type VI CRISPR Cas system and the target protospacer RNA on the transcriptome of *R. capsulatus*, we performed RNA-seq with total RNA samples from the strains *cas13a*^***−***^ and *cas13a*^***+***^, either carrying pCV2_SB6 for ectopic expression of protospacer RNA or without pCV2_SB6. Principle component analysis demonstrated a strong variation of the transcriptome upon simultaneously induced expression of the CRISPR Cas system and protospacer RNA (Figure 5, right panel, green data points) compared to the wild type control with plasmid-borne protospacer expression (blue data points). Interestingly, a global shift of the RNA abundance profile upon expression of protospacer RNA could also be observed in the *cas13a*^**−**^ background (red data points) compared to the wild type, even though this effect was much less pronounced than for *cas13a*^***+***^. The left panel of Figure 5 visualizes the transcriptomic changes observed for cells grown without ectopic expression of protospacer RNA, which are remarkably smaller compared to the strong variations upon simultaneous expression of the CRISPR Cas system and protospacer RNA (right panel).

**Figure 6:**
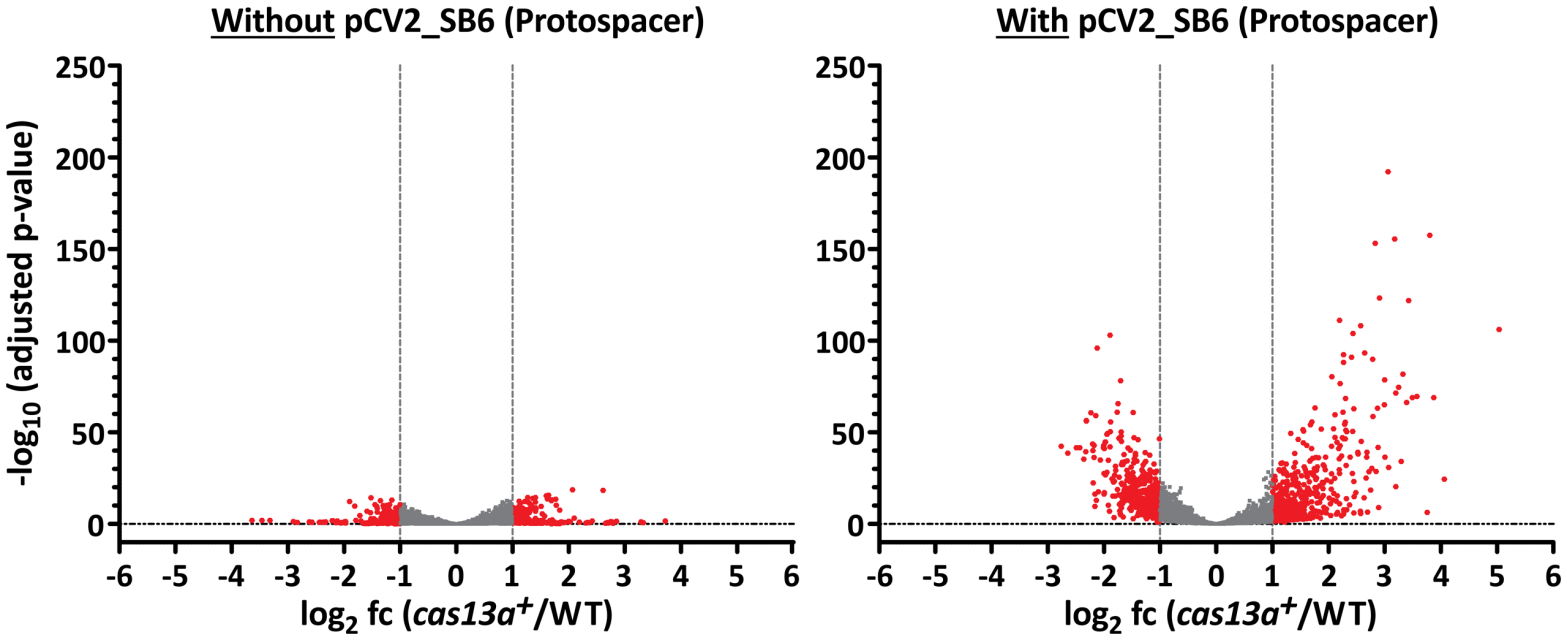
The amount of significantly differentially expressed genes increases upon expression of protospacer RNA. Volcano plots highlighting significantly differentially expressed genes between strain *cas13a*^***+***^ and the *R. capsulatus* wild type. RNAs with an abundance of log_2_ fold change (*cas13a*^***+***^/WT) < 1 and > −1 are marked in gray. The left panel shows RNA-seq data from strains without protospacer sequences, while the right panel shows data from strains carrying pCV2_SB6 for ectopic expression of protospacer RNA. All annotated RNAs (*n* = 3,707) were subject of this analysis.

Volcano plots visualize the strong aberration of global RNA levels, caused by the parallel induction of *cas13a* and ectopically expressed protospacer RNA (Figure 6). While only 200 RNAs showed significantly affected abundance changes (adjusted *p*-value <0.05 and log_2_ fc > 1 or < −1) during induction of *cas13a* in absence of protospacer RNA (left panel), 844 RNAs showed significantly changed abundance (same parameters as before) upon simultaneous induction of *cas13a* and protospacer RNA (right panel) which represents a substantial proportion (~23%) of all annotated RNAs in *R. capsulatus*.

In addition to Figure 6, Table 1 summarizes the amount of significantly affected RNAs upon simultaneous induction of *cas13a* and presence or absence of the protospacer RNA. 5.0% of all genes with adjusted *p*-value <0.05 showed higher and 4.0% lower expression (log_2_ fold change ≥1or ≤ −1) when *cas13a* expression is increased by IPTG in absence of a protospacer. When the protospacer was present, induction of *cas13a* led to much stronger changes of global gene expression levels: 19.3% of all genes with adjusted p-value <0.05 showed higher, 17.8% showed lower expression (log_2_ fold change ≥1 or ≤ −1). Taken together, ~37% of all significantly quantified genes showed more than 2-fold change in their expression, compared to only 9% in absence of the protospacer.

**Table 1:** Amount of significantly differentially expressed genes of strain *cas13a*^***+***^ in presence or absence of protospacer RNA.

*cas13a*^+^/WT	Without protospacer	% of significant RNAs	With protospacer	% of significant RNAs
FDR < 5%	2,181		2,274	
fc ≥ 2	110	5.0	440	19.3
fc ≥ 1.6	351	16.1	634	27.9
fc ≤ −2	88	4.0	404	17.8
fc ≤ −1.6	277	12.7	720	31.7

Global RNA changes (fc: fold change) in strain *cas13a*^***+***^ according to our total RNA-seq based DESeq2 analyses (*cas13a*^***+***^/WT) are shown. Biological triplicates of *cas13a*^***+***^ and wild type, either carrying plasmid pCV2-SB6 (for ectopic expression of protospacer RNA) or without plasmid, were analyzed. The total number of RNAs with indicated fold change as well as the corresponding percentage of significant RNAs (false discovery rate FDR < 5%) are shown. All annotated RNAs (*n* = 3,707) were subject of this analysis.

Based on the fact that the CRISPR Cas system is activated by the simultaneous expression of the system and the presence of protospacer RNA, resulting in the general degradation of cellular RNAs through off-target cleavage, one might have expected a general increase in RNA levels in our transcriptome profiling data. Notably, our library preparation protocol for RNA-seq does not include an RNA fragmentation step to allow bioinformatic mapping of RNA 5′ ends at a native background. Therefore, a higher degree of RNA fragmentation can indeed result in a higher read count per gene. Additionally, it is important to note that a higher fold change in this quantification does not consequently indicate the presence of more intact RNA.

Since, Cas13a cleaves ssRNA in an endoribonucleolytic manner (Shmakov et al., 2015; Abudayyeh et al., 2016) new Cas13a-dependent 5′ sites should arise with higher Cas13a activity. While we could detect a total of 10,086 RNA 5′ end positions within the RNA-seq samples of the *cas13a*^***+***^ strain in presence of the protospacer, only a total of 8,524 RNA 5′ end positions were detected for the wild type in presence of protospacer RNA. From these 10,086 RNA 5′ end positions, 6,399 positions (≈ 63%) were uniquely mapped for samples of *cas13a*^***+***^, while 3,687 RNA 5′ end positions were detected in both the wild type and the *cas13a*^***+***^ background. These numbers (summarized in Supplementary Figure S4) indicate, that in presence of the protospacer not only the total amount of global RNA 5′ ends is increased during elevated *cas13a* expression, but also the distribution pattern of global RNA 5′ ends is thoroughly changed in this strains (as seen by the high amount of uniquely mapped positions compared to the wild type).

**Figure 7:**
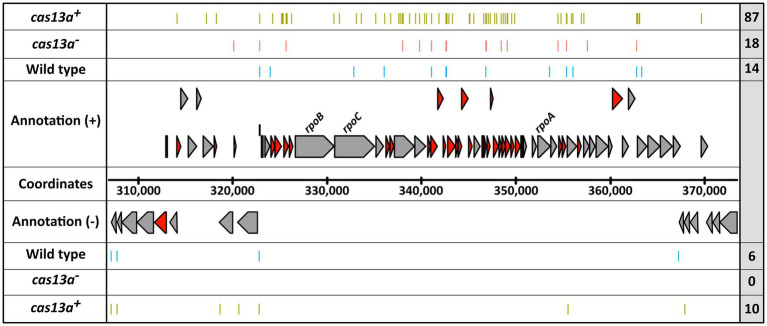
The expression of *cas13a* in presence of protospacer RNA leads to extensive formation of novel RNA 5′ ends within a genomic locus for ribosomal protein encoding mRNAs. The position of RNA-seq detected 5′ ends (colored bars) are visualized for the *R. capsulatus* wild type (blue), the *cas13a*^**−**^ strain (red) and the induced *cas13a*^**+**^ strain (green), each carrying pCV2_SB6 for ectopic expression of protospacer RNA. The 5′ ends obtained from biological triplicates are summarized in each row. The total count of visualized 5′ ends is indicated within the right gray box. mRNAs coding for ribosomal proteins are highlighted in dark red.

Remarkably, several of those 5′ ends uniquely mapped to the strain with elevated *cas13a* expression are clustered in a chromosomal region encoding numerous ribosomal proteins (Figure 7). Within this genomic cluster the total amount of RNA 5′ ends is >6-fold higher upon induced *cas13a* expression compared to the wild type. To validate the accumulation of RNA 5′ ends, ~200 bp fragments including mapped 5′ ends were amplified using qRT-PCR for selected loci within this cluster. These results are shown in Supplementary Figure S5 and confirmed strongly decreased amounts of intact *rplJ*, *rplP* and *rplF* mRNAs for samples of the *cas13a*^***+***^ strain. In addition to the strong accumulation of new RNA 5′ end positions for genes of ribosomal proteins, also three novel RNA 5′ ends were mapped within the ORF *rpoC* and one more within the ORF of *rpoB*, which encode the β subunit and the β′ subunit of the DNA-dependent RNA polymerase, respectively. As ribosomal proteins as well as proper assembly and activity of the RNA polymerase are crucial for growth, fragmentation of these mRNAs (marked in red color in Figure 7) would consequently result in growth inhibition, and thereby could explain the observed growth arrest for cells with increased Cas13a levels in presence of the protospacer (Figure 4). Regulated growth arrest or cell death is frequently employed by bacteria to prevent spreading of phages within the surrounding population (Rousset and Sorek, 2023).

## 4 Conclusion

Beyond an important role in protection of bacteria against phages, alternative roles in physiological functions like stress tolerance, antibiotic resistance, or DNA repair have been described previously for CRISPR Cas systems (reviewed in Ratner et al., 2015). Our results reveal an activation of the CRISPR Cas13a system by various stress factors and especially in late stationary phase in the facultative phototrophic bacterium *R. capsulatus*. The growth arrest that is caused by activation of the Cas13a system is likely to protect the cells against harmful conditions. In contrast to the previous characterization of the *R. capsulatus* CRISPR Cas13a system in *E. coli* (Kick et al., 2022), our data provides interesting insights into the regulation and physiological consequences within its native background. In addition, our results indicate a potential function of this system during Abi similar to the recent analysis of a class 2 type VI system in *L. seeligeri* (Meeske et al., 2020). As it remains unknown by which molecular mechanisms stress factors affect expression of the Cas13a system, future experiments should elucidate these mechanisms and analyze the Cas13a/protospacer-mediated RNA decay in more detail *in vitro* and *in vivo*.

## Data availability statement

The datasets presented in this study can be found in online repositories. The names of the repository/repositories and accession number(s) can be found at: https://www.ncbi.nlm.nih.gov/geo/, GSE255203.

## Author contributions

JK: Writing – original draft, Formal analysis, Investigation, Methodology, Visualization. JB: Formal analysis, Investigation, Methodology, Visualization, Writing – original draft, Validation, Writing – review & editing. TF: Investigation, Methodology, Validation, Visualization, Writing – review & editing. MM: Methodology, Writing – review & editing. TP-K: Methodology, Writing – review & editing, Investigation. FG: Investigation, Writing – review & editing, Validation. JW: Investigation, Writing – review & editing, Resources, Supervision. GK: Funding acquisition, Resources, Supervision, Writing – review & editing, Conceptualization, Writing – original draft.

## Footnotes

^1^ https://github.com/FelixKrueger/TrimGalore
