# Supplementary material for: Function of the RNA-targeting class 2 type VI CRISPR Cas system of Rhodobacter capsulatus
Source: Front Microbiol. 2024 Apr 29;15:1384543. doi: 10.3389/fmicb.2024.1384543 (PMC11089165; doi:10.3389/fmicb.2024.1384543)
Supplement: Supplementary file 1 [file Data_Sheet_1.docx]

Supplementary Material

**Primers, plasmids, and strain construction**

For obtaining the transcriptional fusions of the promoters of the class 2 type VI system (this includes one promoter for each CRISPR array as well as the promoter for the *cas13a* gene), the DNA containing one of the promoters was fused to the ORF for the gene encoding the fluorescent reporter protein mVenus. In the case of the *cas13a* promoter, this was a 307 bp fragment that included the first 41 bp of the coding region of the *cas13a* gene (using primers PCas13a_H_fwd and PCas13a_RBS_X_rev). In the case of the CRISPR array promoters, the size of the DNA region carrying each promoter was 292 bp (CRISPR array 1-4, primers pCRISPR_1-4_short_H_fwd and pCRISPR_1-4_RBS_X_rev) and 296 bp (CRISPR array 5-6, primers pCRISPR_5-6_short_H_fwd and pCRISPR_5-6_RBS_X_rev). These regions started several bp downstream of the transcription start and extended at least 200 nt upstream of the transcription start. In each transcriptional fusion construct, an artificial ribosome-binding site (5′-AAAGGAG-3′) was included in the construct using a linker region located within the reverse primer. Using PCR, this sequence was inserted six nucleotides upstream of the ATG of the gene for mVenus. The promoter::mVenus fusions were carried on the low copy plasmid pPHUmVenus, previously used as a fluorescence reporter construct in *Rhodobacter sphaeroides* (McIntosh et al., 2019). pPHUmVenus was based on the plasmid pPHU231, originally developed for monitoring gene expression in *R. capsulatus* (Hübner et al., 1991). The three pPHUmVenus-derived plasmids are referred to in this study as pPHU-P*cas13a*-mVenus, pPHU-P1-4-mVenus, and pPHU-P5-6-mVenus (see Table S3).

The *R. capsulatus* mutant strains (Fig. S2) were generated via the suicide vector pk18mob2 (Schäfer et al., 1994) carrying a specially designed transcription-control cassette which has been previously described (Kretz et al., 2023). This transcription-control cassette, also known as ACIT (Alphaproteobacteria chromosomally integrating transcription-control cassette), was developed to insert into the chromosome via homologous recombination. Following the insertion into the chromosome, ACIT controls transcription of a gene of interest that is close to the site of insertion. Transcription control is performed using two inducers: crystal violet for transcriptional repression and IPTG for transcriptional activation. As preparation for ACIT control over the transcription of the *cas13a* gene (3858 bp), a 431 bp fragment was amplified via PCR from the *R. capsulatus* genome. For the PCR, two primers were used: Cas13a_GOI_X_fwd, which binds at 23 bp upstream of the *cas13a* ATG start codon, and Cas13a_GOI_K_rev, which binds at 408 bp downstream of the ATG start codon. The 431 bp fragment was cloned into the ACIT cassette (pK-ACIT 2.0) to serve as a guide for the homologous recombination (plasmid pK-ACIT 2.0-*cas13a*, see Table S2). Following homologous recombination, transcription from the native promoter of *cas13a* was terminated by a transcription terminator from the cassette. Furthermore, an IPTG inducible promoter, a specialized component of the ACIT cassette, now controls the full-length *cas13a* gene (inducible *cas13a*, see Fig. S2B). Addition of crystal violet (0.1 µM) activates production of the LacI repressor protein, blocking transcription from the IPTG inducible promoter, whereas addition of IPTG (0.5 mM) releases the promoter from LacI and thus induces *cas13a* expression. In addition, a variant of the ACIT cassette (pK-ACIT 1.2-*cas13a*) was used which lacks the IPTG inducible promoter, thereby serving as a no-transcription control, both in the presence and absence of IPTG and crystal violet. To create this variant (*cas13a****^-^***, see Fig. S2A), the 431 bp fragment was also cloned into a version of the ACIT cassette (pK-ACIT 1.2) which lacks the IPTG inducible promoter.

To allow for the overexpression of a protospacer, the protospacer from the CRISPR array 5-6 (ACGATTGCCCTTATCCTCAATGAGACCCGGCA) was initially included into the reverse primer for amplifying via PCR the promoter region of *cas13a*. Thereby, the *cas13a* promoter was fused to the protospacer, and this fusion was followed by a downstream-located synthetic RBS and the gene for mVenus after being cloned into the reporter plasmid pPHUmVenus. However, this plasmid construct (named pPHU_PS6) appeared to hinder growth of *R. capsulatus* (following the selection of colonies with pPHU_PS6 on agar via tetracycline). We suspected that the expression of the protospacer from the *cas13a* promoter was too strong to support growth. Therefore, an additional cloning step was performed in order to bring the expression of the protospacer under the control of IPTG induction. For the cloning, the protospacer, synthetic RBS and mVenus were amplified from pPHU_PS6 as a single PCR product and inserted into plasmid pCV2, a medium copy plasmid (based on the pBBR replicon) previously developed for controlled expression in alphaproteobacteria (Kretz et al., 2023). The primers used for this step were SB6_X_fwd and mVenus_K_rev, creating the pCV2 derivative pCV2_SB6. Furthermore, to test whether the protospacer was functional, a variant was created (pCV2_SB6mut) using primers SB6mut_X_fwd and mVenus_K_rev. This variant contained a single nucleotide A → C exchange within the protospacer. These constructs were conjugated into *R. capsulatus* strains (WT, *cas13a****^-^*** and inducible *cas13a*). Here, it is important to note that the presence of pCV2_SB6 in the *R. capsulatus* inducible *cas13a* strain meant that the expression of not only *cas13a* but also the protospacer RNA were IPTG inducible.

For the fusion of the Flag tag to Cas13a, the oligos Sp_RBS_3F_fwd and K_X_3F_rev, in an initial step, were annealed to each other and ligated within the ACIT cassette. This ensured that the Flag-tag encoding sequence was present in the ACIT cassette, together with an RBS and an ATG translation start. Importantly, these oligos also carried XbaI/KpnI restriction digest sites, which allowed, in a second step, the cloning of the first 408 bp of the coding region of *cas13a* using primers GOI_Cas13a_F_X_fwd and Cas13a_GOI_K_rev (Table S2). Following integration of this version of the ACIT cassette (pK-ACIT 2.0-F-*cas13a*) into the chromosome, *Flag*::*cas13a* expression was under the control of the inducible promoter.

For construction of the RpoE deletion strain the primer pairs f_up_00698 / r_up_00698 and f_down_00698 / r_down_00698 were used to amplify the fragments 00698up and 00698down which were ligated into plasmid pDrive (Qiagen). The Ω-Km cassette (*Bam*HI fragment from plasmid pHP45Ω-Km (Fellay et al., 1987) was then inserted into the *Bam*HI site between the up and down fragments and the *Eco*RI-*Hind*II fragment was isolated and inserted into pPHU281 (Hübner et al., 1991) to generate plasmid pPHU00698. This plasmid was transferred into strain SB1003 by conjugation (Klug and Drews, 1984) and by selecting for kanamycin resistant and tetracycline sensitive colonies the Δ*rpoE* strain was isolated.

**Table S1.** *Rhodobacter* strains used in this study. Antibiotic resistances against spectinomycin (Sp^R^) and kanamycin (Km^R^) are indicated.

| *Rhodobacter capsulatus* strains | Description | Source |
| --- | --- | --- |
| SB1003 | Wild type | Yen and Marrs et al., 1976 |
| Δ*rpoH*I | SB1003 with disrupted *rpoH_I_*, Sp^R^ | Mercer and Lang, 2014 |
| Δ*rpoHII* | SB1003 with disrupted *rpoH_II_*, Km^R^ | Mercer and Lang, 2014 |
| Δ*rpoE* | SB1003 with disrupted *rpoE,* Km^R^ | This study |
| *cas13a^+^* | SB1003 with inserted pK-ACIT 1.2-*cas13a* suicide plasmid | This study |
| *cas13a^-^* | SB1003 with inserted pK-ACIT 2.0-*cas13a* suicide plasmid | This study |

**Table S2.** Primers used in this study.

| Name | Sequence |
| --- | --- |
| pCas13a_H_fwd | GCATAAGCTTGCCGCTCCAGTGGCAATTAC |
| pCas13a_RBS_X_rev | GATCTCTAGACTCCTTTGAATTCGCTGATCGTCCGCCC |
| pCRISPR_1-4_short_H_fwd | GATCTCTAGAGTGATGTGAGGCTCAGGTCCC |
| pCRISPR_1-4_RBS_X_rev | GATCTCTAGACTCCTTTGTGATGTGAGGCTCAGGTCCC |
| PCRISPR_5-6_short_H_fwd | GATCAAGCTTGATCCGCGAAGATGCCATCATC |
| pCRISPR_5-6_RBS_X_rev | GATCTCTAGACTCCTTTGGTGATGTGCTCCTTTACCACTGG |
| Cas13a_GOI_X_fwd | GTTACATGCCTCTAGAGGTTTTGTGCTGGGGGCG |
| Cas13a_GOI_K_rev | GTTACAGATCGGTACCGGGCGGATCCGCTTCGAATTT |
| SB6_X_fwd | GATCTCTAGAAATTACGATTGCCCTTATCCTCAATGAG |
| SB6mut_X_fwd | GATCTCTAGAAATTACGATTGCCCTTATCCGCAATGAG |
| mVenus_K_rev | CATAGGTACCTTACTTGTACAGCTCGTCCATGC |
| PS6_X_fwd | GATCTCTAGAAATTACGATTGCCCTTATCCTCAATGAG |
| PS6mut_X_fwd | GATCTCTAGAAATTACGATTGCCCTTATCCGCAATGAG |
| f_up_00698 | ACTAGAATTCAAGGCGACCGGATCAGAT |
| r_up_00698 | ACTAGGATCCAAATCGGGATCGGTCGCG |
| f_down_00698 | CACCGGATCCCCGGGAATTCCCGCCAGCGCGATACGTCC |
| r_down_00698 | TCTAGAGCGCAAACTTTTCGGCCTC |
| Sp_RBS_3F_fwd | GATCACTAGTAACCTTGGGGATTGAAGAATGGATTACAAGGATCACGATGGT |
| K_X_3F_rev | GATCGGTACCAGTTGGCGTCTAGACTTGTCATCGTCATCCTTGTAATCG |
| GOI_Cas13a_F_X_fwd | GATCTCTAGAATGCAGATTGGCAAGGTTCAAGG |
| Northern probe for CRISPR1-4 RNA | TCAGTCCGCCGTCGTCTTGGCGG |
| Northern probe for 5S rRNA | CTTGAGGACGCAGTACCATTG |
| qRT_*rplJ*_fwd | CGAAAATGCGTGACGTGGGTG |
| qRT_*rplJ*_rev | GCTTTGACACCGGCCTTGTC |
| qRT_*rplP*_fwd | GCAACCGAAACGGACGAAATTCC |
| qRT_*rplP*_rev | GTTTGGAGGTCACCGGCACA |
| qRT_*rplF*_fwd | GCAAGAAACCGGTCGAAATGCC |
| qRT_*rplF*_rev | CTCTTTCTTGAAGCCGGTGGAGA |
| qRT_*sinI*_fwd | CATCGCGTAATCACGCA |
| qRT_*sinI*_rev | GGTGCTGGCTGCGAC |

**Table S3.** Plasmids used in this study.

| Name | Derived from | Reference | Resistance | Use in this study |
| --- | --- | --- | --- | --- |
| pPHU-P*cas13a*-mVenus | pPHUmVenus | This study | tetracycline | transcriptional fusion |
| pPHU-P1-4-mVenus | pPHUmVenus | This study | tetracycline | transcriptional fusion |
| pPHU-P5-6-mVenus | pPHUmVenus | This study | tetracycline | transcriptional fusion |
| pK-ACIT 2.0-*cas13a* | pK-ACIT 2.0 | This study | kanamycin | inducible *cas13a* strain |
| pK-ACIT 1.2-*cas13a* | pK-ACIT 1.2 | This study | kanamycin | *cas13a****^-^*** strain |
| pK-ACIT 2.0-F-*cas13a* | pK-ACIT 2.0 | This study | kanamycin | inducible *flag*::*cas13a* strain |
| pCV2_SB6 | pCV2 | This study | gentamycin | inducible protospacer |
| pCV2_SB6mut | pCV2 | This study | gentamycin | inducible mutated protospacer |





**Supplementary Figure 1.** *cas13a* promoter activity in stationary phase in the wild type and in mutants for alternative sigma factors. (**A**) The normalized fluorescence intensity of indicated *R. capsulatus* strains carrying the transcriptional reporter plasmid pPHU-P*cas13a*-mVenus is shown. Fluorescence intensity and OD_660_ were measured after 8 h (white bars) or 24 h (gray bars) of growth. The mean value and standard deviation of biological triplicates is plotted. (**B**) The normalized fluorescence intensities of pPHU-P*cas13a*-mVenus carrying strains was measured during exponential phase (white bars) and after stress induction for 1 h (gray bars). Stress was induced by addition of either 0.005% (w/v) SDS + 1 mM EDTA, 1 mM tBOOH or 10 mM H_2_O_2_. The mean value and standard deviation of biological triplicates are plotted. Student's two-sided t-test was used to assess the statistical significance of the difference in mean values (***: p-value < 0.001).





**Supplementary Figure 2.** Genomic modification of the *cas13a* controlling promoter allows inducible expression of *cas13a*. Schematic overview of the modified locus from the *cas13a****^-^*** strain (**A**), and the inducible strain *cas13a* strain (**B**). While addition of crystal violet (CV) represses the *cas13a* promoter, addition of IPTG leads to induction. Both strains were generated via single homologous recombination with a suicide plasmid, leading to substitution of the native *cas13a* promoter. (**C**) Western blot for Cas13a protein levels of the inducible *cas13a* strain (*cas13a****^+^***) in presence of crystal violet (0.1 µM) or IPTG (0.5 mM). Independent biological triplicates of the modified strains and duplicates of the wild type (WT) grown either for 5 h or 48 h after addition of CV or IPTG were analyzed.





**Supplementary Figure 3.** Altered expression of *cas13a* shows only minor effects on stress sensitivity, and growth of *R. capsulatus* under non-stress conditions. (**A**) Growth analysis of the *R. capsulatus* strains with modified *cas13a* expression systems under non-stress conditions. To monitor growth, the optical densities (λ = 660 nm) of wild type, *cas13a****^-^***, or inducible *cas13a* (either with or without IPTG-induction) cultures were measured for 56 h. The mean value of biological triplicates is shown. (**B**) Zone of inhibition assay for screening of altered stress sensitivities. Softagar plates containing wild type, *cas13a^-^*, or inducible *cas13a* cells (either with or without IPTG-induction) were treated with 5 µl droplets of indicated stress inducing agents. The diameter of the resulting zone of inhibition was measured after 48 h of growth at 32°C. Used stressor concentrations were as following: 1 M H_2_O_2_, 5% (w/v) SDS + 1 mM EDTA, or 10 mM methylene blue. The mean value and standard deviation of biological triplicates is plotted. Student's two-sided t-test was used to assess the statistical significance of the difference in mean values of biological triplicates of each strain compared to wild type (n. s.: not significant; *: p-value < 0.05; **: p-value < 0.01).


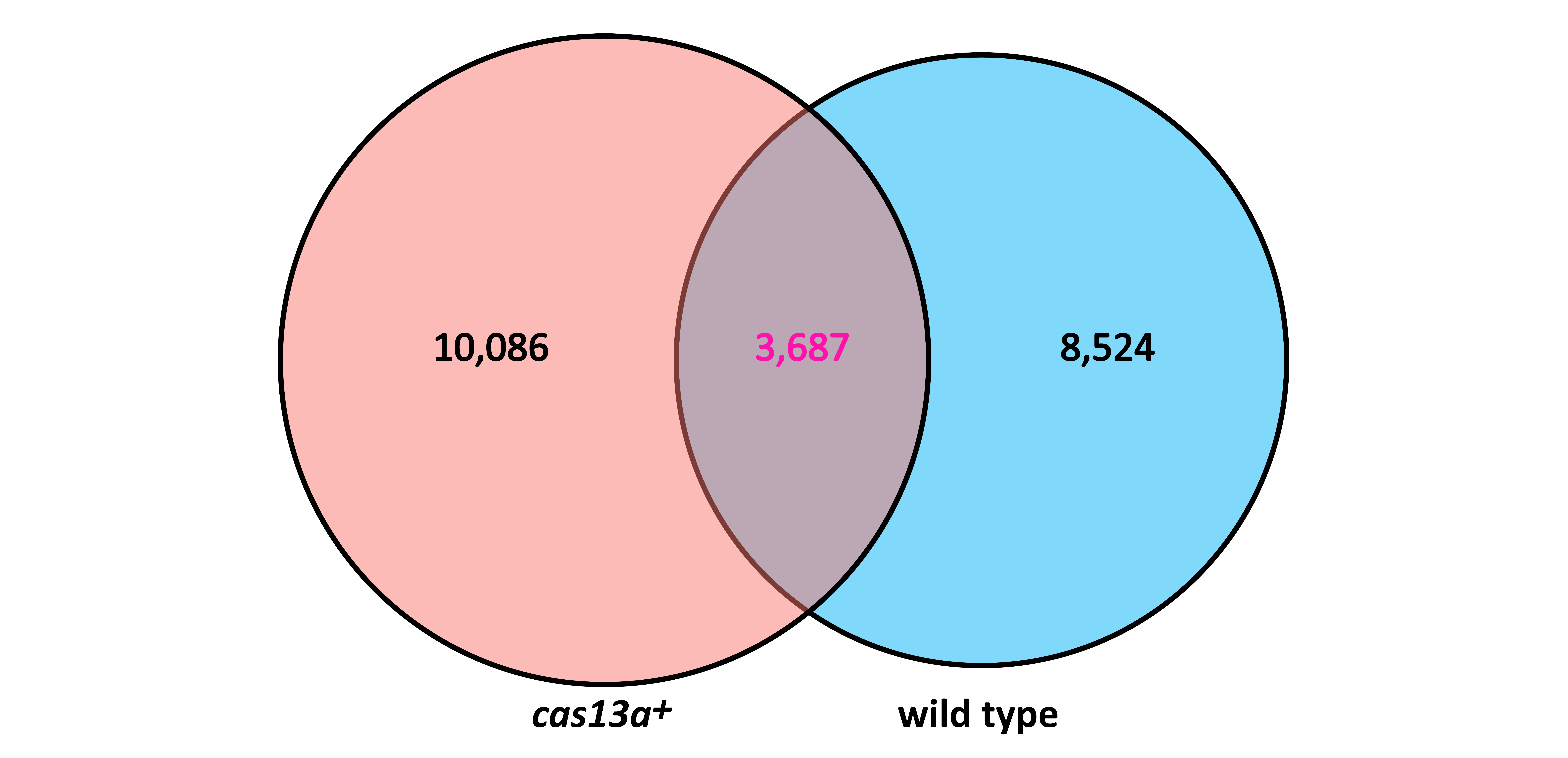


**Supplementary Figure 4.** The Venn diagram shows the amount of detected RNA 5′ end positions for the *cas13a****^+^*** (red) and wild type (blue) strain, both carrying pCV2_SB6. The amount of identical RNA 5′ end positions detected for both strains is indicated in magenta.


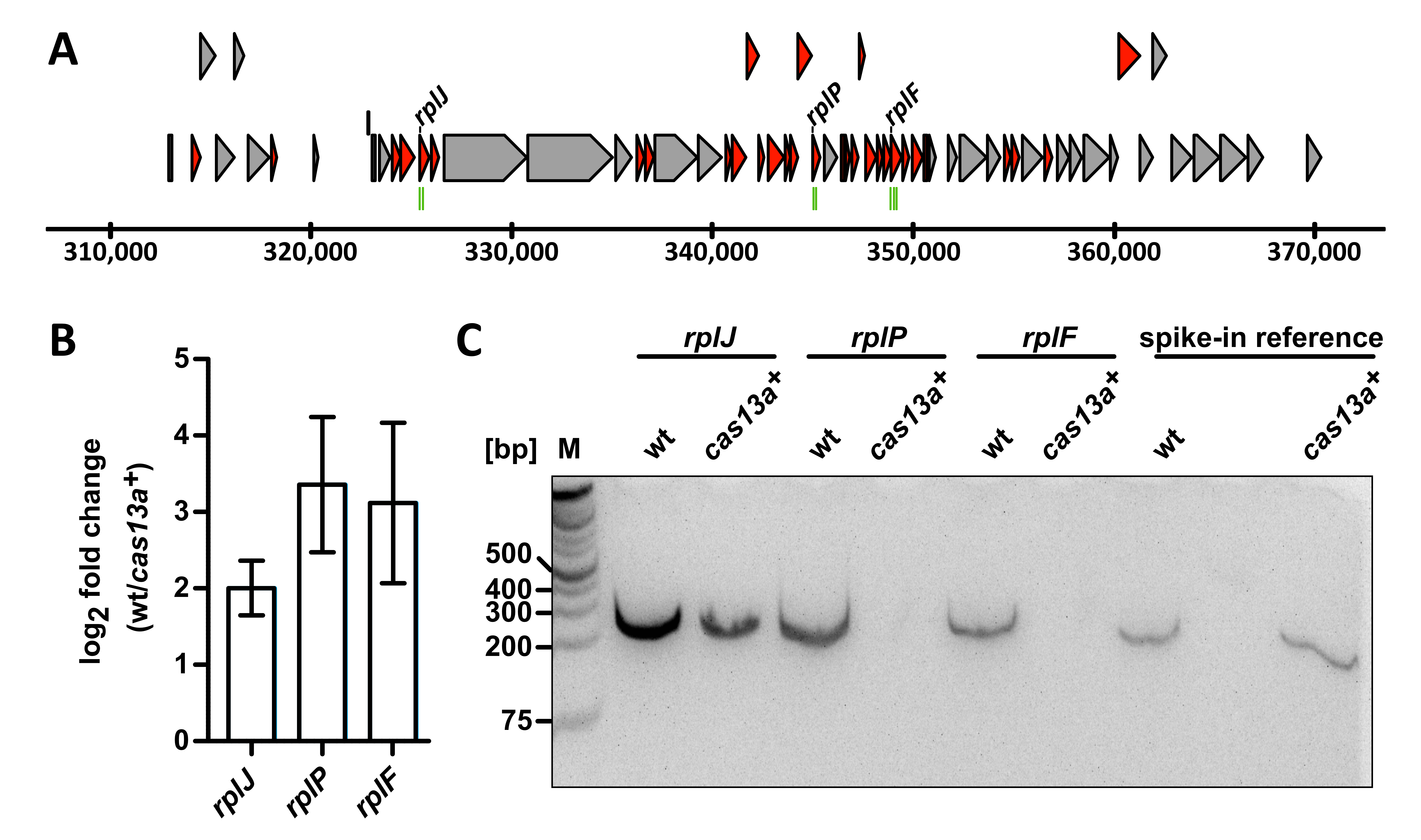


**Supplementary Figure 5.** qRT-PCR amplification of selected loci with mapped RNA 5′ end. (**A**) Overview showing the genomic cluster with high density of ribosomal protein encoding genes. Genes for ribosomal proteins are marked in red. Highlighted fragments of *rplJ*, *rplP* and *rplF* with uniquely mapped RNA 5′ ends in the *cas13a****^+^*** strain carrying pCV2_SB6 (green) were subject to qRT-PCR analysis. (**B**) Spike-in qRT-PCR with DNA-free total RNA extracted from the wild type and the *cas13a****^+^*** strain, both carrying pCV_SB6. The relative abundance of the tested mRNA regions is shown. The standard deviation of the mean value of biological triplicates is indicated as error bar. Primers used for the qRT-PCR analysis are listed in Table S2. (**C**) 10 µl of the resulting qRT-PCR products were electrophoretically separated on a 10% polyacrylamide gel. 5 µl of GeneRuler 1 kb Plus DNA ladder (Thermo Scientific) were loaded as length standard. Relevant reference bands and their corresponding length (bp: base pairs) are marked. DNA bands were stained with ethidium bromide and subsequently visualized under a UV light.
